# Functional Expression of an Orchid Fragrance Gene in *Lactococcus lactis*

**DOI:** 10.3390/ijms13021582

**Published:** 2012-02-02

**Authors:** Adelene Ai Lian Song, Janna O. Abdullah, Mohd Puad Abdullah, Norazizah Shafee, Raha A. Rahim

**Affiliations:** 1 Department of Cell and Molecular Biology, Faculty of Biotechnology and Biomolecular Sciences, University Putra Malaysia, 43400, UPM Serdang Selangor, Malaysia; E-Mails: adelene_song@yahoo.com (A.S.); puad@biotech.upm.edu.my (P.A.); 2 Department of Microbiology, Faculty of Biotechnology and Biomolecular Sciences, University Putra Malaysia, 43400, UPM Serdang, Malaysia; E-Mails: janna@biotech.upm.edu.my (J.O.A.); nshafee@biotech.upm.edu.my (N.S.); 3 Institute of Bioscience, University Putra Malaysia, 43400, UPM Serdang, Malaysia

**Keywords:** *Vanda* Mimi Palmer, *Lactococcus lactis*, isoprenoids, sesquiterpene synthase, orchid, fragrance

## Abstract

*Vanda* Mimi Palmer (VMP), an orchid hybrid of *Vanda tesselata* and *Vanda* Tan Chay Yan is a highly scented tropical orchid which blooms all year round. Previous studies revealed that VMP produces a variety of isoprenoid volatiles during daylight. Isoprenoids are well known to contribute significantly to the scent of most fragrant plants. They are a large group of secondary metabolites which may possess valuable characteristics such as flavor, fragrance and toxicity and are produced via two pathways, the mevalonate (MVA) pathway or/and the 2-C-methyl-D-erythritol-4-phosphate (MEP) pathway. In this study, a sesquiterpene synthase gene denoted *VMPSTS*, previously isolated from a floral cDNA library of VMP was cloned and expressed in *Lactococcus lactis* to characterize the functionality of the protein*. L. lactis*, a food grade bacterium which utilizes the mevalonate pathway for isoprenoid production was found to be a suitable host for the characterization of plant terpene synthases. Through recombinant expression of VMPSTS, it was revealed that VMPSTS produced multiple sesquiterpenes and germacrene D dominates its profile.

## 1. Introduction

Isoprenoids are a large and diverse class of naturally occurring metabolites. While all organisms produce isoprenoids such as prenyl lipids for cell membrane synthesis and ubiquinone for electron transport as part of their primary metabolism [1], many also produce isoprenoids as secondary metabolites with valuable characteristics such as flavor, fragrance, toxicity and medicinal properties. These qualities have been widely applied to pharmaceuticals such as the anti-malarial artemisinin and the chemotherapeutic paclitaxel, nutraceuticals such as carotenoids, flavors such as limonene and linalool, fragrances such as citronellol and geraniol [2] and fuels such as farnesane [3]. Isoprenoids have been reported to be produced via two pathways, the mevalonate (MVA) pathway and/or the 2-C-methyl-D-erythritol-4-phosphate (MEP) pathway, depending on the organism. Both pathways converge at a common precursor, isopentenyl pyrophosphate and its isomer dimethylallyl diphosphate (IPP/DMAPP) [4]. These precursors make up the C_5_ isoprenes which form the backbone structure for all isoprenoids. Depending on the number of C_5_ isoprene units, isoprenoids are further divided into mono-, sesqui-, di-, tri- and tetraterpenes [5].

There are more than 50,000 known isoprenoids to date and the major source of these isoprenoids is plants [6]. Unfortunately, since isoprenoids are produced as secondary metabolites in plants, they are not produced in appealing amounts in many cases or have to be extracted from a complex matrix. This combined with other limiting factors such as long life cycles, seasonal and regional limitations and the need for complicated down-stream processes to isolate the isoprenoid compounds have made plants to be unfavorable commercial isoprenoid factories. For example, a single dose of Taxol^®^, a chemotherapeutic drug produced from the diterpene taxadiene, requires the entire bark of a 100 year old *Taxus brevifolia* tree to produce [7]. In view of this, heterologous host production is deemed the savior to these limitations and much effort has been put into producing plant isoprenoids in microbial cell factories. Since prokaryotes possess the necessary metabolic pathways involved in isoprenoid production, plant terpene synthases cloned into prokaryotes enable them to produce plant isoprenoids *in vivo*. This can be carried out by utilization of the host’s precursors such as geranyl pyrophosphate (GPP) and farnesyl pyrophosphate (FPP) for the production of mono- and sesquiterpenes respectively, provided that the pool of precursors is sufficient to maintain the requirements of both the endogenous pathways as well as the fabricated one. Therefore, efficient production hosts such as *Escherichia coli* [8,9] and yeasts [10–12] have been metabolically engineered for increased heterologous isoprenoids production.

In recent years, *Lactococcus lactis*, a Gram-positive lactic acid bacterium, which has been used for centuries in the food industry, has found new and exciting applications and has been developed into cell factories for the production of bioactive compounds such as enzymes, peptides and vaccine antigens [13,14]. Compared to *E. coli*, *L. lactis* has certain advantages as a heterologous host such as being food-grade and thereby classified as GRAS (Generally Regarded as Safe) and the absence of inclusion bodies, enabling uncomplicated product recovery [15]. Coupled with the development of the nisin-controlled gene expression (NICE) system [16] which uses the food grade inducer nisin for expression, *L. lactis* has become one of the most successful Gram-positive hosts for genetic engineering. However, to date, there are only a handful of plant genes which has been expressed in *L. lactis* [17–19], of which only one is an isoprenoid gene, the linalool/nerolidol synthase (FaNES) from strawberry (*Fragaria x ananassa*) [20].

In the current paper, we report on the functional expression of a sesquiterpene synthase gene from *Vanda* Mimi Palmer (VMP) in *L. lactis* which uses the mevalonate pathway for isoprenoid production, unlike most prokaryotes which use the MEP pathway. VMP is an award winning ornamental orchid hybrid of *Vanda* Tan Chay Yan and *Vanda tessellate* [21]. This orchid hybrid is known for its distinct sweet fragrance which was found to be dominated by a concoction of benzenoid, phenylpropanoid and terpenoid compounds [22]. The VMP sesquiterpene synthase gene, designated as *VMPSTS* was previously isolated and molecularly characterized based on its sequence but was not functionally identified (NCBI GenBank Accession no: **EU145743**) [23]. In the current study, through recombinant protein expression of the VMPSTS protein in *L. lactis*, the production of the plant isoprenoids by the recombinant sesquiterpene synthase was analyzed and identified. More importantly, *L. lactis* was shown to be a potential host for heterologous isoprenoid production, specifically sesquiterpenes, concurring with findings by Hernandez *et al*. [20].

## 2. Results and Discussion

### 2.1. Plasmid Construction and Stability of Recombinant Strains

The sesquiterpene synthase from *Vanda* Mimi Palmer, previously isolated by Chan *et al*. [23] was successfully cloned into pNZ8048 yielding pNZ:VMPSTS. Sequencing of the gene of interest showed 98% identity with **EU145743**. This recombinant plasmid was then transformed into *L. lactis* NZ9000 host cells and was found to be 100% stable by growing the strains in the absence of antibiotics for 100 generations and subsequently streaking out 100 random colonies on plates supplemented with 7.5 μg/mL chloramphenicol. Plasmid integrity was further confirmed by restriction enzyme digestion analysis using *Pst*I and *Xba*I (data not shown).

### 2.2. Expression of VMPSTS in *L. lactis*

**Figure 1:**
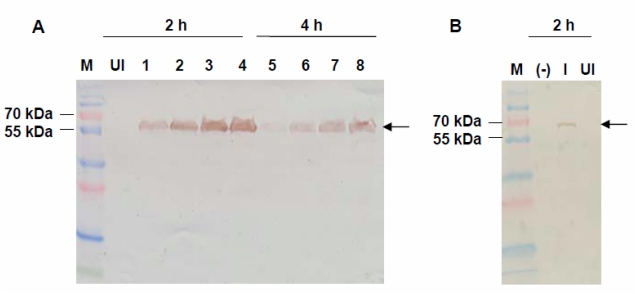
Western blot of VMPSTS expression profiles showing the expected band size of 63 kDa. (**A**) Clones harboring pNZ:VMPSTS were grown and induced with nisin at different concentrations and durations. Lanes 1 and 5: 10 ng/mL nisin; 2 and 6: 20 ng/mL nisin; 3 and 7: 40 ng/mL nisin; 4 and 8: 60 ng/mL nisin; (**B**) Comparison between induced, uninduced and negative control samples. M: PageRuler™ Prestained Plus Protein Ladder; (−): Empty pNZ8048 plasmid; I: 40 ng/mL nisin for 2 h; UI: uninduced.

**Figure 2:**
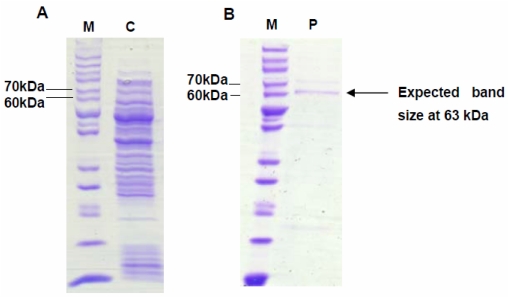
SDS-PAGE analysis showing crude (**A**) and his-tag purified (**B**) protein extracted from clones harboring the pNZ:VMPSTS plasmid. Clones were grown and induced with 40 ng/mL nisin for 2 h. Lane M: PageRuler™ Unstained Protein Ladder; C: Crude total protein extract; P: His-tag purified VMPSTS protein at 63 kDa.

Expression of VMPSTS in *L. lactis* was analyzed by SDS-PAGE and Western blot analyses. In the crude protein extracts comparing induced and uninduced cultures for pNZ:VMPSTS as well as the negative control (clones harboring empty pNZ8048 plasmid), the SDS-PAGE did not show any distinct bands corresponding to an expected protein size of 63 kDa which was predicted to be exclusively visible in the induced culture (data not shown). However, subsequent analysis by Western Blot showed a clear expected band size of 63 kDa which was only present in the induced cultures but not in the uninduced cultures or the negative control (Figure 1). This shows that the recombinant protein was successfully expressed in *L. lactis* but the protein of interest may be masked by other host proteins of the same size in the crude protein extracts when seen on the SDS-PAGE gel. Optimization of the induction conditions showed that *VMPSTS* expression can be induced using 10–60 ng/mL nisin with no distinct differences in expression from 40 ng/mL nisin onwards, based on the intensities of the bands in the Western blot. However, 2 h induction was preferable compared to 4 h as VMPSTS expression seemed to decrease or perhaps degrade as the induction time was increased. Since growth rates of induced and uninduced cultures were similar (data not shown), this could possibly be associated with the production of isoprenoids *in vivo* which have been shown to follow exponential growth and to cease at the stationary phase owing to the FDP substrate being produced during growth for primary metabolism [24]. Therefore in subsequent studies, 40 ng/mL nisin was used to induce expression for 2 h prior to harvesting the cells. Purification of the crude protein using a Ni-NTA column to bind the N-terminal histidine tag of the recombinant protein produced clear protein band of the expected size of 63 kDa as observed through SDS-PAGE analysis confirming successful expression of the recombinant plant terpene synthase in *L. lactis* (Figure 2).

### 2.3. Enzymatic Assay of Crude VMPSTS

**Figure 3:**
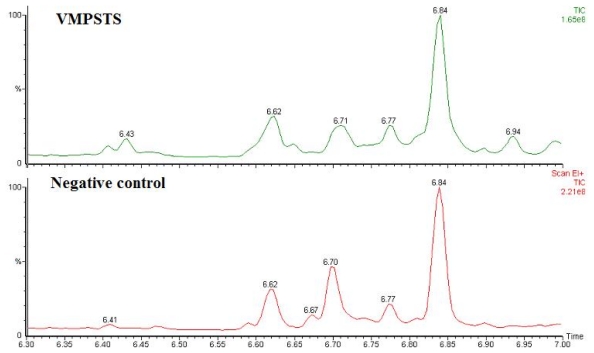
GC-MS chromatogram of products formed by crude VMPSTS protein at different retention times (RT). Negative control used consisted of NZ9000 host harboring empty pNZ8048 plasmid. At RT 6.00 min to 7.00 min: for VMPSTS, at 6.43: copaene, 6.62: farnesene, 6.71: aromadendrene, 6.77: 2-tridecanone, 6.84: germacrene D and 6.94: δ-cadinene were observed while in the negative control at 6.62: nerylacetone, 6.67: 4,6-di-tert-butyl-m-cresol, 6.70: 1-dodecanol, 6.77: 2-tridecanone and 6.84: methyl 4,4,7-trimethyl- 4,7-dihydroindan-6-carboxylate were noted.

Functionality of the recombinant VMPSTS of both crude and purified recombinant protein extracts were analyzed by enzymatic assay using two different assay buffers. However, both buffer systems yielded comparable results with no significant differences. When the crude proteins of VMPSTS and the negative control were used for the enzymatic assay, majority of the FPP were converted to farnesol which gave the highest peak (data not shown) potentially by non-specific endogenous phosphatases, even when sodium tungsten and sodium fluoride were added to the assay as phosphatase inhibitors. Phosphatases have been reported to metabolize FPP to produce farnesol [25]. Apart from the non-specific phosphatase activity on FPP, it was reported that FPP was prone to non-enzymatic hydrolysis to nerolidol and farnesol when Mg^2+^ was present whereas in the presence of Mn^2+^, the hydrolysis product was almost exclusively nerolidol [26]. These cations are common co-factors for enzymes including terpene synthases and hence, precaution should be taken to rule out possibilities of products formed from such non-enzymatic hydrolysis of FPP in the terpenoid product profiles. Indeed, this was also observed in our study when FPP was incubated with the assay buffer without any protein extracts. Due to the high amount of farnesol which was produced when using crude protein extracts, only trace amounts of other sesquiterpenes which may be true products of VMPSTS were detected with peak areas <6% of the farnesol peak (Figure 3). Copaene and δ-cadinene peaks were observed at retention times (RT) 6.43 min and 6.94 min respectively and exclusively in assays performed with crude VMPSTS (Figure 3). Although peaks at RT 6.62 min, 6.71 min and 6.84 min observed from the VMPSTS crude protein also corresponded to sesquiterpene products and were identified as farnesene, aromadendrene and germacrene D respectively, peaks with similar retention times were also present in the negative control. Interestingly, a comparison of the negative control mass spectra to the NIST and Wiley libraries identifies the peak at RT 6.62 min as nerylacetone in comparison with farnesene in the VMPSTS crude protein and negates both peaks at RT 6.70 min and 6.84 min as sesquiterpenes, but identifies them as 1-dodecanol and methyl 4,4,7-trimethyl-4,7-dihydroindan-6-carboxylate instead.

### 2.4. Enzymatic Assay of His-Tag Purified VMPSTS and End Product Analysis

**Figure 4:**
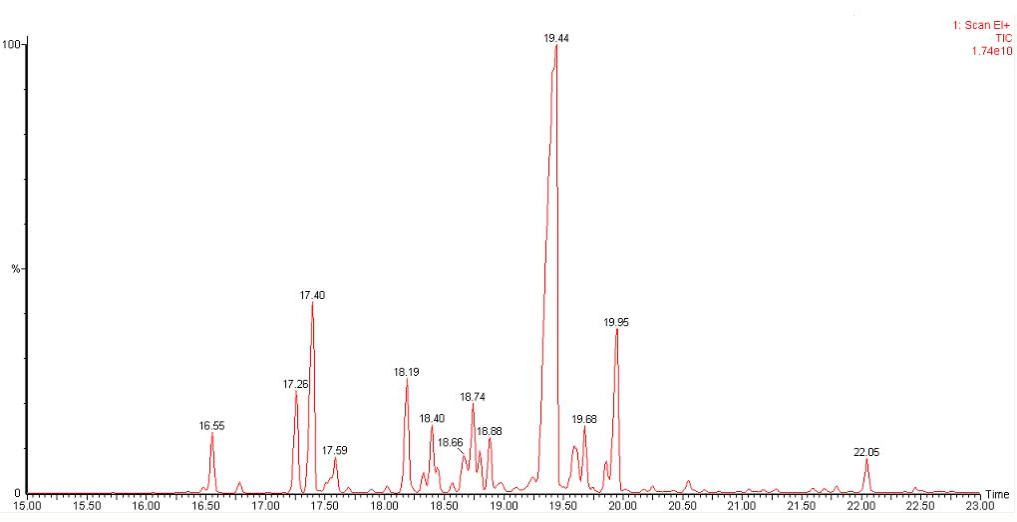
GC-MS chromatogram showing sesquiterpenes produced from purified VMPSTS protein. The peaks in this chromatogram are listed in Table 1.

**Figure 5:**
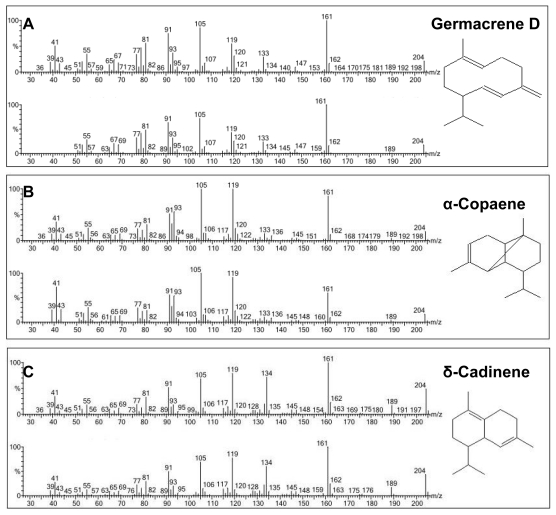
Mass spectra of top three sesquiterpenes produced by recombinant VMPSTS in comparison with the corresponding mass spectra from the NIST or Wiley Library. Top panel shows mass spectra of the sample compound while bottom panel shows mass spectra of the compound in the library. (**a**) is germacrene D; (**b**) is α-copaene; and (**c**) is δ-cadinene.

Further clarification on the identities of the products produced was made by enzymatic assays analysis using His-tag purified VMPSTS proteins. Consistent with the previous crude VMPSTS result, germacrene D was still the major product comprising 48.9% of the total sesquiterpenes produced while α-copaene and δ-cadinene contributed 9.5% and 8.8%, respectively (Figure 4). The mass spectra of the top three major products obtained based on peak area are shown in Figure 5. A host of other minor sesquiterpenes, undetected using the crude extract, was also produced albeit at lesser amounts when using the purified VMPSTS (Table 1). However, farnesene and aromadendrene which were detected in the crude protein extract analysis were not present in the purified protein analysis, suggesting that these could be intermediate metabolites or their derivatives resulting from the endogenous conversion of FPP to farnesol. Apart from the major peak at RT 19.44 min which was identified to be germacrene D, three other peaks at RT 18.19 min, 18.40 min and 18.66 min also had similar mass spectra and conferred germacrene D as the highest hit. This could represent four different stereoisomers of germacrene D which is not surprising considering germacrene D has 2 chiral centers in its carbon backbone. Furthermore, germacrenes have two endocyclic double bonds at 1(10)- and 4(5)- positions which could result in four geometric isomers [27]. It should also be noted that germacrene sesquiterpenes are prone to Cope rearrangements induced by GC-MS conditions such as elevated temperature resulting in artefacts [27]. As such, the ß-elemene peak at RT 17.56 min produced by the purified VMPSTS protein may not be a true product of VMPSTS. A few other sesquiterpenes were also produced but in negligible amounts (below 1%) and/or had match scores below 800 and thus were deemed ambiguous and are not reported here. Terpene synthases capable of producing multiple terpene products are very common due to rearrangements and quenching of the intermediate carbocations products [28,29]. The types of sesquiterpenes found in our research were in agreement with the reported *ab initio* investigation of the cyclization of germacrene D which is usually accompanied by a host of cadinane and muurolane sesquiterpenoids [30]. All peaks were identified by comparison with the NIST and Wiley libraries only since standards for these specialized sesquiterpenes are not easily available. However, comparison of retention times with standards and isolation of the major compounds followed by NMR spectroscopy are suggested to be carried out as future work for structure determination which will strongly confirm the identities of the peaks identified by the NIST and Wiley libraries.

Previously, it was reported that nerolidol was the only sesquiterpene detected at 0.5% of the total scent, when analyzed directly from open VMP flowers using SPME sampling [22]. Copaene and germacrene D were also found in the essential oils of VMP at 0.17% and 0.13% respectively when the compounds from open VMP flowers soaked in hexane overnight were analyzed using GC-MS [31]. The terpenoid composition obtained directly from the open flowers of VMP differs slightly from the products produced by the *in vitro* recombinant VMPSTS study. One salient observation was that cadinene, ylangene, muurolene and muurolol, caryophyllene, elemene, and gurjunene found in the *in vitro* study were not detected either in the scent or the essential oils of VMP open flowers. This could be due to the low amounts produced in the plants or the sampling method used which could subject the major sesquiterpenes to chemically rearrange in different ways than in the *in vitro* studies, resulting in different compositions of minor sesquiterpene compounds. In addition, types of hosts may also influence composition of products [32]. There are also many other terpenes found in VMP open flowers especially monoterpenes which contribute to the fragrance of the plant. However, the terpene synthases responsible for these terpenoids have not been isolated and characterized yet.

When the VMPSTS protein sequence was subjected to a BLAST search, α-humulene (synonym α-caryophyllene), germacrene-D and δ-cadinene terpene synthases were among the top hits with up to 48% identity, along with other unidentified sesquiterpene synthases. This shows that the protein sequence of VMPSTS is closely related to similarly functional sesquiterpene synthases from other plants such as ginger (*Zinger zerumbet* and *Zinger officinale*) and castor oil plant (*Ricinus communis*). Although germacrene D synthases has been isolated and characterized from other plants such as ginger (*Zingiber officinale*) [29] and grapevine (*Vitis vinefera*) [33], both enzymes and the one used in this study displayed different product compositions. The germacrene D synthase isolated from *Zingiber officinale* produced germacrene D and germacrene B as its major product along with nine co-products including germacrene C, α-humulene and *trans*-nerolidol. The germacrene D synthase from *Vitis vinefera* on the other hand only produced germacrene D as a major product and δ-cadinene as its minor product. The differences in the product compositions of germacrene D synthases from different plants demonstrate the vast array of isoprenoid variation and the versatility of the terpene synthases producing them.

In terms of bioactivity, germacrene D has been reported to have insecticidal effects against mosquitoes [34] and to repel aphids [35]. However, it is an attractant to the Tobacco Mudworm Moth, *Heliothis virescens* [36]. Alpha-copaene has been specifically identified as a potent attractant of male Mediterranean fruit flies, *Ceratitis capitata* [37] while δ-cadinene has been shown to have antileishmanial activity against the protozoan parasite, *Leishmania* which causes the human disease, leishmaniasis [38].

To date, there has been much work done on functional identification of various types of novel plant terpene synthase from sunflower (*Helianthus annuus*) [39], ginger (*Zingiber officinale*) [40], oregano (*Origanum vulgare*) [41], grapes (*Vitis vinefera*) [28] and others which involve functional identification of the terpene synthase via *in vitro* assays and GC-MS analyses of the gene products in the *E. coli* system. However, due to the tremendous variety, versatility and potential of these secondary metabolites, we have only begun to tap into an enormous warehouse of possibilities and these efforts are done with the hope of discovering novel terpenoids with high industrial and economic value. Increased production of these terpenoids in heterologous hosts via metabolic engineering is being highly pursued. The knowledge that all the 50,000 known terpenoids originate from the same common precursor IPP/DMAPP opens up the possibility of engineering one successful platform which can be used for the production of any of those 50,000 terpenoids. While *E. coli* and *S. cerevisiae* are the most common heterologous hosts for the optimization and production of isoprenoids, this study supports previous research by Hernandez *et al*. [20] that the GRAS *L. lactis* is another potential host for heterologous isoprenoid production.

## 3. Experimental Section

### 3.1. Bacteria Strains and Growth Conditions

*L. lactis* NZ9000 strain [42] which is a nisin-negative derivative of *L. lactis* MG1363 was used in this study. NZ9000 has the n*is*R and *nis*K genes from the nisin gene cluster inserted into its chromosome. These genes enable nisin induction of the P_nisA_ promoter on the pNZ8048 plasmid which was used for all *L. lactis* cloning purposes in this study. All *L. lactis* strains were cultured in M17 broth or agar [43] supplemented with 0.5% (w/v) glucose (GM17) and 7.5 μg/mL chloramphenicol whenever necessary. *L. lactis* was typically grown at 30 °C as a standstill culture. When screening for *L. lactis* transformants, M17 agar supplemented with 0.5% (w/v) glucose, 0.5 M sucrose and 7.5 μg/mL chloramphenicol was used. Top 10 *E. coli* strain (Invitrogen, CA, USA) was grown in LB broth and cultured at 37 °C with shaking at 250 rpm. When screening for *E.coli* transformants, LB agar supplemented with ampicillin (100 μg/mL) and 40 μg/mL of 5-bromo-4-chloro-3-indolyl-β-D-galactopyranoside (X-gal) was used.

### 3.2. VMPSTS Gene Amplification and Plasmid Construction

The sesquiterpene synthase gene from VMP used in this project has 3 different nucleotides at 616A > G, 734G > A and 1560C > T compared to the NCBI deposited VMP gene (Accession no: **EU145743**) [23]. The primers used to amplify the *Vanda* sesquiterpene synthase gene were 5′-CT CTGCAGAA*CATCACCATCACCATCAC*ATGGAGACTCTCAAAGC-3′ (forward) and 5′-CTTCTAGA CTACATAGAGTTAGAAATATCAG-3′ (reverse) using the cDNA library generated from previous work [23]. The restriction enzymes (underlined) used for directional cloning were *Pst*I and *Xba*I and the His-tag sequence which was incorporated into the forward primer is italicized. The PCR reaction mixture contained 1× reaction buffer, 2 mM MgCl_2_, 0.2 mM dNTP, 5 units of Taq polymerase, 0.5 μM of forward and reverse primers and approximately 20 ng of template DNA. The reaction was run for 30 cycles using a 94 °C for 1 min, 60 °C for 1 min and 72 °C for 2 min temperature-time profile. The amplified gene was cloned into pGEM-T Easy Vector (Promega, WI, USA) and transformed into Top 10 *E. coli* competent cells prior to sub-cloning into the *L. lactis* plasmid, pNZ8048 yielding pNZ:VMPSTS which was then transformed into the *L. lactis* NZ9000 host. The heat-shocked method was used for transforming *E. coli* competent cells [44] while the electroporation method was used for transforming *L. lactis* competent cells [45]. The transformed *E. coli* recombinants were screened by blue-white screening as well as ampicillin resistance selection while the *L. lactis* transformants were screened based on chloramphenicol resistance selection. Positive transformants were confirmed by colony PCR, double digestion with *Pst*I and *Xba*I and sequencing.

### 3.3. Expression of VMPSTS in *L. lactis*

For protein expression, overnight culture of *L. lactis* NZ9000 harboring pNZ:VMPSTS or empty pNZ8048 (as negative control) were inoculated into fresh GM17 medium at 5% (v/v) and grown to an OD_600_ of 0.4. The culture was split into sub-samples consisting of uninduced samples and samples induced with nisin at concentrations ranging from 10–60 ng/mL for 2 and 4 h. For subsequent experiments, cultures were induced at 40 ng/mL for 2 h after determining the optimum induction condition. After induction, the cells were harvested in an assay buffer (15 mM 3-morpholino-2- hydroxypropanesulfonic acid (MOPSO), pH 7, 10% v/v glycerol, 10 mM MgCl_2_, 1 mM MnCl_2_, 1 mM sodium ascorbate and 2 mM dithiothreitol) [20] or (20 mM Tris-HCl, pH 8.0, 5 mM MgCl_2_ and 2 mM dithiothreitol), modified from Chen *et al*. [46], by centrifugation at 1438 × g for 10 min at 4 °C. Crude extracts were prepared by subjecting the cells to sonication treatment using the Omni Ruptor 4000 (Omni International, GA, USA) set to 10% power and pulsed for 2 min per sample. Then, the samples were centrifuged at 16,000 × g, for 10 min, at 4 °C with the final aqueous phase subjected to SDS-PAGE and Western Blot analyses.

For recombinant protein purification, His SpinTrap (GE Healthcare, WI, USA) columns equilibrated with ten column volumes of binding buffer containing 20 mM imidazole were used according to the manufacturer’s protocol to trap recombinant protein via the N-terminal His-tag fusion. Subsequently, the column was washed with another ten column volumes of binding buffer and the final elution was performed in the presence of 300 mM imidazole. After purification, the proteins were desalted using HiTrap Column (GE Healthcare, WI, USA) according to the manufacturer’s protocol. At each step, the protein was analyzed by SDS-PAGE and Western Blot. SDS-PAGE analysis was done according to Laemmli [47] while Western Blot was performed using the chromogenic Western MAX™ horse radish peroxidase (HRP) kit (Amresco, OH, USA) according to the manufacturer’s protocol with slight modifications. The primary antibody used was mouse anti-his IgG (Novagen, NJ, USA) while the secondary antibody, HRP conjugated goat anti-mouse IgG, was supplied in the kit. Briefly, the samples were run on 12% SDS-PAGE Tris/glycine gels and transferred to polyvinylidene fluoride (PVDF) membrane using a semi-dry blotter (Biorad, CA, USA). Blocking was done using 1% (w/v) bovine serum albumin. PageRuler™ Unstained Protein Ladder (Fermentas, Ontario, Canada) was used as SDS-PAGE marker while for Western Blot, PageRuler™ Prestained Plus Protein Ladder, Fermentas, Ontario, Canada) was used.

### 3.4. Enzymatic Assays

Enzymatic assay for the recombinant sesquiterpene synthase was performed as previously described [46,20]. Briefly, the crude or purified protein extract in the assay buffer was mixed with 20 μL farnesyl diphosphate substrate from a 1 μg/μL stock (Sigma Aldrich, MO, USA) in a total volume of 1 mL. Phosphatase inhibitors, 0.4 mM NaWO_4_ and 0.2 mM NaF, were also added in the reaction mixture. The mixture was then incubated with shaking at 30 °C for 3 h and the reaction was stopped with 1 mL of 4 M CaCl_2_. All the above reactions were performed in a 20 mL headspace vial which was then subjected to solid phase microextraction (SPME) sampling using a 100 μm polydimethylsiloxane (PDMS) coated fiber. Extraction time used was 15 min at 60 °C and desorption time was 5 min. Blank tests were run in between sampling runs to ensure no carryover from the SPME fiber or the GC-MS system.

### 3.5. GC-MS Analysis

Products from the enzymatic assays were analyzed using the GC-MS Turbomass Clarus 600 (Perkin Elmer, UK) and the GC column used was Perkin Elmer Elite 5 MS (30 m × 0.25 mm ID). The GC oven conditions were from an initial temperature of 40 °C (1 min hold) to 250 °C (3 min hold) with a 35 °C ramping temperature increase per min. For purified VMPSTS, ramping time was decreased to 7 °C per min for better separation as the former ramping rate was found to be unable to resolve the minor peaks satisfactorily. The column flow was held constant at 1 mL/min. The injection port was maintained at 250 °C, the transfer line at 220 °C while the MS source temperature was maintained at 200 °C. The MS, operated at 70 eV was set to scan from *m/z* 35 to 400. Products were identified by comparing the spectra to those in the National Institute of Standards and Technology (NIST) library 2008 and the Wiley Registry of Mass Spectral Data, 8^th^ Edition. The cut-off match score for the mass spectra of the products compared to the NIST and Wiley library used were >700 for the crude protein and >800 for the purified protein.

## 4. Conclusions

In this project, the *VMPSTS* gene previously isolated from the orchid hybrid, *Vanda* Mimi Palmer has been functionally identified to produce germacrene D, copaene and δ-cadinene among others. This was achieved by cloning and expression of the enzyme in *L. lactis* followed by *in vitro* enzymatic assays to identify the sesquiterpene products produced via GC-MS analysis. Based on the major sesquiterpene produced, VMPSTS is identified to be a germacrene D synthase. To our knowledge, this is the first terpene synthase gene from the *Orchidaceae* family to be functionally identified through recombinant technology and only the second plant isoprenoid gene ever to be cloned and expressed in *L. lactis*.

## Figures and Tables

**Table 1:** Summary of retention times, match score and percentages of sesquiterpenes produced.

Sesquiterpenes	Retention Time	Match score	Percentage
δ-Elemene	16.55	950	2.4
Ylangene	17.26	955	4.5
α-Copaene	17.40	943	9.5
ß-Elemene	17.56	922	2.2
Germacrene D	18.19	903	5.3
Germacrene D	18.40	913	3.7
Germacrene D	18.66	890	2.1
α-Gurjunene	18.74	905	4.2
ɛ-Muurolene	18.80	824	1.2
α-Caryophyllene	18.89	946	2.1
Germacrene D	19.44	966	48.9
γ-Muurolene	19.68	881	2.2
γ-Cadinene	19.86	934	1.3
δ-Cadinene	19.95	945	8.8
.tau.-Muurolol	22.05	927	1.6
